# Association between Lack of Social Support from Partner or Others and Postpartum Depression among Japanese Mothers: A Population-Based Cross-Sectional Study

**DOI:** 10.3390/ijerph17124270

**Published:** 2020-06-15

**Authors:** Akito Yamada, Aya Isumi, Takeo Fujiwara

**Affiliations:** Department of Global Health Promotion, Tokyo Medical and Dental University, Tokyo 113-8519, Japan; akito.com1@gmail.com (A.Y.); isumi.hlth@tmd.ac.jp (A.I.)

**Keywords:** postpartum depression, social support, partner, maternal mental health, Japan

## Abstract

Lack of social support is a known risk factor for postpartum depression (PPD). However, the association between lack of social support from a partner or others and PPD remains unknown. We examined this association among Japanese mothers. We distributed an original questionnaire to mothers participating in a three- or four-month health check-up program over October to November 2012 in Aichi Prefecture, Japan. Of the 9707 eligible mothers, 6590 responded to the questionnaire (response rate: 68%). Social support from a partner or others was assessed based on whether the mother can consult with her partner or others (i.e., parents, relatives, and friends who are close by or far) on childcare. PPD was assessed with the Edinburgh Postnatal Depression Scale. The data were analyzed using multiple logistic regression analysis for four categories: no social support from either a partner/others, social support from a partner only, social support from others only, and social support from both, adjusted for possible covariates. Mothers who have no social support from either a partner/others, have social support from a partner only, and have social support from others only were 7.22 (95% confidence interval [CI], 1.76–29.6), 2.34 (95% CI, 1.37–3.98), and 3.13 (95% CI, 2.11–4.63) times more likely to show PPD, respectively, in comparison with mothers who have social support from both, after adjustment of possible covariates. Mothers with no social support from a partner, but have social support from others, showed significant risk for PPD, which may be invisible. Further prevention effort is needed to detect PPD cases, with a focus on mothers without support from their partner.

## 1. Introduction

Postpartum depression (PPD) is a serious mental disorder with a prevalence of approximately 10–15% worldwide [1,2,3]. Women with PPD often have marked distress and dysfunction that disrupt their daily life [4,5], and are more likely to have subsequent depression and suicide [6,7]. Moreover, PPD can impair maternal–infant relationships and psychosocial development of the children [8,9,10,11]. Given the prevalence and the long-term implications for women and their children, PPD represents a public health concern [12].

Recent studies indicated several risk factors for PPD, including low levels of social support, stressful life events, childcare stress, low income and marital dissatisfaction [13,14,15]. Of these factors, social support has been considered both as one of the strongest predictors of PPD and a target of psychosocial intervention [5,13]. In fact, several studies showed poor perceived social support was related to PPD [16,17,18,19,20].

Adult attachment style is one of the major determinants of levels of social support [21]. Women with insecure attachment, who are more likely to have lower levels of social support from a partner and others such as parents or friends, are known to be at higher risk of PPD than those with secure attachment [22,23]. Furthermore, women who have a partner’s support, but do not have others’ support, might also have insecure attachment style [21], whereas women who have others’ support, but who do not have a partner’s support, might have secure attachment style, but have poor marital relationship quality. Considering the adult attachment style theory [24] and previous studies regarding marital relationship [25], having social support from a partner but not from others, or vice versa, may have differential impact on PPD. However, the association of each of the combinations of levels of partner’s support and those of others’ support with PPD has not been investigated.

Furthermore, although it can be assumed the partner is the closest person to the mother, the relevance of social support from a partner for PPD has been rarely explored. The risk of PPD in mothers who lack a partner’s support, but have support from others, is often overlooked, since they receive support from several sources other than their partner, such as parents and friends. Therefore, mothers who have support from others, but not from their partner, may be an important high-risk group. To date, there are few studies that have indicated that both a partner’s support and friends’ and family’s support are independently associated with a lower risk of PPD [25,26,27,28]. Thus, using a population-based sample, we investigated the association between lack of social support from a partner or others and PPD in Japan.

## 2. Materials and Methods

### 2.1. Samples

Participants were recruited from Aichi Prefecture, Japan. All 54 municipalities in Aichi were asked to participate in this study, and 45 municipalities agreed, which resulted in 80% coverage of the total population in the prefecture. The target subjects were all mothers (N = 9707) who were registered in a three- or four-month health check-up program for their children between October and November 2012, in participating municipalities. In 34 municipalities, an anonymous questionnaire was mailed directly to the target mothers before the health check-up program, and answered questionnaires were collected at the health check-up site. In the remaining 11 municipalities, the questionnaires were distributed at the check-up sites and the participants sent completed questionnaires by mail to each health center. In total, the participation rate for the three- or four-month health check-up in Aichi Prefecture was 97.9%, and 6590 women responded to the questionnaire (response rate; 68%). Single mothers (i.e., unmarried, divorced, widowed or other) (n = 104) and mothers with missing responses to questions about marital status or social support (n = 395) were excluded from the sample, resulting in a sample size of 6195 mothers.

This study was approved by the Ethics Committee of the National Center for Child Health and Development (code: 611). The Ethics Committee approved that personal informed consent could be exempted if participants responded anonymously to the questionnaire.

### 2.2. Assessment of Social Support from Partner or Others

The questionnaire assessed women’s social support regarding the number of persons they can consult about childcare and whether mothers can consult with their partner, parents, relatives, and friends who are close by or far away, respectively. The following questions were asked (in Japanese): “How many relatives or friends can you consult with about childcare?” and “If you have someone to consult, who can you consult with? Please circle ‘Yes’ or ‘No’ based on whether or not you can consult with for each of the following person(s): partner, your parents or your partner’s parents, relatives (including your brothers or sisters, or your partner’s brothers or sisters), friends who are close by, and friends who are far away”. Mothers who can consult with their partner were defined as “mothers having social support from partner”. Likewise, mothers who can consult with at least one person other than their partner (i.e., parents, relatives, or local or distant friends) were defined as “mothers having social support from others”.

### 2.3. Screening for PPD

The Japanese version of the Edinburgh Postnatal Depression Scale (EPDS), a 10-item self-report questionnaire, was used to evaluate PPD [29]. Items were rated on a four-point scale to produce a summative score ranging from 0 to 30, with lower scores indicating better maternal mood. The reliability and validity of the EPDS have been well documented in multiple languages. Using a cut-off score of ≥9, the EPDS has 75% sensitivity, which is defined as the proportion of people with the disease who will have a positive result, and 93% specificity, defined as the proportion of people without the disease who will have a negative result [30]. Therefore, the cut-off point of 9 was used in this study.

### 2.4. Covariates

The questionnaire also assessed the parental characteristics, gestational and perinatal characteristics, maternal history of intimate partner violence and history of mental disorders. Parental demographics include maternal and paternal age, whether expectant mothers had someone who supported them during pregnancy (yes/no), subjective economic status (i.e., stable, able to manage, difficult to manage, or unstable), maternal and paternal employment status (i.e., working full-time, working part-time, or not working), cohabitation status (i.e., living with infant’s grandparents or not). Gestational and perinatal characteristics include having infertility treatment (yes/no), feeling when they noticed the pregnancy (i.e., felt happy, felt happy although pregnancy was unexpected, muddled because it was unexpected, puzzled, had no feelings), sex of the child (i.e., boy, girl), birth order (i.e., first child, subsequent child), delivery satisfaction (yes/no), low birth weight of the child (<2500 g), preterm birth (<37 weeks), maternal smoking and drinking history during pregnancy (yes/no), history of miscarriage and termination of pregnancy (yes/no). Maternal history of intimate partner violence includes verbal and physical violence (i.e., none, a few times, sometimes, often). Maternal history of mental disorders includes whether women had depressive symptoms during one year prior to pregnancy and whether women had previously been diagnosed with a mental disorder or had received treatment for a mental disorder before getting pregnant. Age difference between parents was calculated by subtracting maternal age from paternal age. As for subjective economic status, “difficult to manage” and “unstable” were collapsed for the analysis, since a low percentage of participants answered “unstable” (2.2%) [31]. The responses on feelings when they noticed the pregnancy of “muddled because it was unexpected”, “puzzled” and “had no feeling” were collapsed to “unintended pregnancy” due to a low percentage of participants answering “muddled because it was unexpected” (6.1%), “puzzled” (0.2%) and “had no feeling” (0.2%), and these feelings were related to unintended pregnancy [32]. Similarly, for verbal and physical intimate partner violence, “a few times”, “sometimes” and “often” were collapsed, since a low percentage of participants answered “sometimes” (3.7%, 0.3%) and “often” (0.5%, 0.1%) [33].

### 2.5. Statistical Analysis

The associations between possible risk factors and PPD were analyzed using multiple logistic regression, especially regarding four categories of mothers, (i.e., mothers having both a partner’s and others’ support, mothers with only a partner’s support, mothers with only others’ support, and mothers having neither a partner’s nor others’ support). In addition to an initial bivariate model, adjusted models were employed. Model 1 was adjusted for demographic and social covariates, including parental demographics (maternal age, age difference between parents, someone who supported them during pregnancy, subjective economic status, employment status for mothers and fathers, and living with infant’s grandparents). Model 2 was further adjusted statuses for covariates in model 1 plus gestational and perinatal covariates (infertility treatment, whether pregnancy was planned, sex of the child, birth order, delivery satisfaction, low birth weight, preterm, maternal smoking history, maternal drinking history, and history of miscarriage, and termination of pregnancy). Model 3 was adjusted for covariates in model 2 plus both verbal and physical intimate partner violence. Finally, model 4 was adjusted for covariates in model 3 plus mental health status (maternal depressive symptoms during one year prior to pregnancy and maternal history of mental disorder). Paternal age was not used due to multicollinearity with maternal age (r = 0.71). As a reference group, a category with the lowest risk of PPD or that with the largest number of participants in each variable was used. A *p*-value of <0.05 was considered to be statistically significant. All analyses were performed using the STATA/SE 13.1 software package (STATA Corporation, College Station, TX, USA).

## 3. Results

Participant characteristics are presented in Table 1. The mean of maternal and paternal age was 31.4 and 33.3 years, respectively. Of the 6195 women, 134 (2.2%) reported that they did not have someone who supported them during pregnancy. Regarding subjective economic status, 659 (11%) reported their finances were “difficult to manage” or “unstable”. As for the infants, 522 (8.4%) and 682 (11%) of them were living with grandfather and grandmother, respectively. A total of 726 (12%) mothers had undergone infertility treatment. When mothers were notified of their pregnancy, 443 (7.2%) felt muddled or puzzled, or had no feelings. Regarding intimate partner violence, 617 (10%) and 60 (1.0%) mothers experienced verbal and physical violence from their partner, respectively. There were 867 (14%) mothers who suffered from depressive symptoms in the year prior to pregnancy and 86 (1.4%) had a history of mental disorders.

As shown in Table 2, 170 (2.7%) mothers reported they did not consult with their partner, indicating no social support from a partner, and 119 (1.9%) mothers reported they did not consult with either their parents, relatives, or friends close by or far, also indicating no social support from others. Mothers were classified into four categories based on social support from partner and others. A total of 5908 (95.4%) mothers had both their partner’s and others’ social support; 108 (1.7%) had their partner’s social support, but did not have that of others; 168 (2.7%) had others’ social support, but did not have that of their partner; 11 (0.2%) had no social support from either their partner or others. The distribution of the EPDS score showed that 536 (8.7%) women scored 9 or above, which indicates that those women were considered to have PPD.

Table 3 shows the bivariate and multivariate association between each type of mother and PPD. In the crude model, mothers with social support from their partner but no support from others, and those with support from others but not from their partner, and those who had no social support from their partner nor others were 3.32 (95% confidence interval [CI], 2.07–5.32), 7.18 (95% CI, 5.17–9.97), and 21.5 (95% CI, 6.27–73.7) times more likely to have PDD, respectively, in comparison with mothers that had social support from both. After controlling for parental, gestational and perinatal characteristics and history of intimate partner violence and mental disorders, mothers with their partner’s support only were 2.34 (95% CI, 1.37–3.98) times more likely to have PPD, compared with mothers that had support from both. In addition, mothers with others’ support only were 3.13 (95% CI, 2.11–4.63) times more likely to have PPD. Furthermore, mothers with neither a partner’s nor others’ support were 7.22 (95% CI, 1.76–29.6) times more likely to have PPD, suggesting that the lack of a partner’s support and others’ support is independently associated with the risk of PPD.

## 4. Discussion

To the best of our knowledge, this is the first study investigating the association between social support from a partner and others, such as grandparents or friends, and PPD using a large number of subjects in Japan. This study found that the lack of either social support from a partner or others showed higher risk of PDD in mothers, compared with those who have social support from both a partner and others. Furthermore, mothers that do not have social support from either a partner or others showed high risk of PPD, that is, 7 times more likely to have PPD.

Several studies reported that a lack of support from a partner was associated with PPD [26,27], and others have reported that a lack of support from both a partner and others was associated with PPD [25,28]. However, none of them analyzed the interaction between a lack of partner’s support and others’ support for the risk of PPD. Hence, we analyzed this interaction as a risk factor for PPD, and found that mothers with others’ social support but not having that of the partner was a high-risk group for PPD (p for interaction = 0.99). This finding is important because mothers who have support from others, but not from their partner, were not usually considered as high-risk mothers for PPD due to the availability of social support from several sources.

There may be several possible mechanisms between the lack of support from a partner and PPD. First, parenting stress can be higher among a nuclear family, thus, expectation for support from a partner might be high, resulting in a lack of it posing more stress, which can be a risk of PDD. In fact, among the families raising child(ren) in Japan in 2017, 82.7% of them were nuclear, whereas only 14.2% were living with grandparent(s) [34]. In nuclear families, the partner is thought to be the only person who can understand the situation and to assist the mother at home. Second, mothers who did not have support from their partner may have preoccupied attachment style, and thus, they are not good at developing interpersonal relationships, including asking for support from their partner [35]. Previous studies found that preoccupied attachment style is associated with depression [35]. Third, based on marital relationship theory, poor marital relationship itself was associated with increased stress in the postpartum period and risk of PPD [13]. Therefore, poor marital relationship may be a confounding factor between not having support from a partner and PPD. Additional studies are necessary to reveal the mechanism between a lack of support from a partner and PPD.

Our results also showed that mothers who did not have support from others have higher risk of PPD. Several mechanisms between a lack of support from others and PPD can be considered. First, relative stress due to comparison with other mothers might be a risk for PPD, and support from others can mitigate the relative stress. For example, if they were unable to breastfeed, they may blame themselves and lose their confidence as a mother. Mothers who have support from relatives or friends share their challenges and difficulties in childcare and receive comfort and encouragement. This reassurance can be considered as a protective factor against PPD. Therefore, mothers without support from others may have a higher risk of PPD than those with support from others. Second, mothers with insecure adult attachment, especially avoidant attachment style, tend to have lower levels of support from others. Thus, mothers with avoidant attachment style are thought to have higher risk of PPD. In fact, several studies identified insecure attachment style as a risk factor for PPD [22,23]. Further studies are needed to explore how a lack of support from others influence the risk of PPD.

Our results also revealed that mothers without support from either their partner or others had a higher risk of PPD than those with support from both. The OR of mothers without any support tended to be higher than that of mothers with their partner’s support only or that of mothers with others’ support only, although the difference did not reach statistical significance (*p* = 0.14 and 0.26 in model 4, respectively). This might imply that a lack of support from a partner and others independently increased the risk of PPD, and those who do not have support from a partner or others can be considered as the highest risk group in terms of social isolation.

In addition, our results also showed that both young mothers aged <25 years and old mothers aged ≥40 years were associated with PPD (Table 3). Young mothers might be sensitive to infant care stress, such as infant crying [36]. Older mothers might have more burden of family obligations and less vitality than other mothers [37]. These might be correlated with higher risk of PPD in younger (<25 years) and older (≥40 years) mothers. In contrast, birth order and parental employment status were not associated with PPD (Table 3), which is inconsistent with a previous study reporting that primiparity was found to be risk factors for PPD [38]. The discrepancy might be due to sampling method, i.e., current study employed population-based sampling, while the previous study used hospital-based convenience sampling.

The present study has several limitations. First, it is not possible to determine the causality between a lack of social support from a partner or others and PPD due to a cross-sectional design. It is possible that depressed mothers tend to evaluate levels of social support more negatively than non-depressed mothers. Thus, longitudinal studies with more frequent measurements of both levels of social support and PPD are required to estimate the causality. Second, although the EPDS is a well-validated screening scale [30], it is not the clinical criteria for PPD. Third, in this study, the presence of social support was assessed based on whether mothers can consult with partner and/or others, such as relatives and friends. This questionnaire was originally created to capture the types of social support based on social network index measuring social connections, such as contacts with their spouse, children, relatives and friends [39]. In previous studies, the levels of social support were assessed by the Social Provisions Scales, the Japanese version of the Social Support Questionnaire, Multidimensional Scale of Perceived Social Support or original questionnaires [18,25,28,40]. Because these measurements captured the level of social support and not the source of social support, we decided not to use these validated measures of social support but the originally created social support, although not validated, instead. Future studies may use both levels and sources of social support using validated scales. Fourth, instrumental social support from the partner and/or others, such as provision of transportation, was not examined in this study. Fifth, other possible risk factors for PPD, such as stressful life event/s and genetic vulnerabilities, were not assessed in this study.

Despite these limitations, the current study provides insights into the protective effects of social support on PPD. In Japan, approximately 8–15% of mothers experience PPD [41,42]. Japanese mothers tend to spend more time on infant care than mothers in western countries [43], which may imply that they are prone to having devastating stress from infant care [44]. Considering the fact that more than 80% of families raising child(ren) were nuclear [34] and lack of social support is one of the strongest risk factors of PPD [5,13], social support from a partner or others might be important to prevent PPD. In Japan, the majority of pregnant women register their pregnancy at public health centers and local municipal governments provide home-visit programs to families with infants via public health centers [45]. In addition, most mothers with infants participate in a three- or four-month health check-up program. Through this system, it might be desirable that public health nurses ask mothers about the levels of social support from their partner or others they are receiving in order to assess the risk of PPD. If mothers lack social support from a partner or others, public health nurses can establish a close relationship with such mothers, so that mothers become able to consult with them about infant care and maternal mental health. Furthermore, fathers might be encouraged to provide enough support for their partner and to participate actively in infant care activities to prevent the mother from becoming depressed.

## 5. Conclusions

In conclusion, a lack of social support from a partner or others, which is defined as women not being able to consult with their partner or others, increases the risk of PPD. Additional longitudinal research, including more frequent assessment of these factors and PPD, is needed to determine the causality between these risk factors and PPD.

## Figures and Tables

**Table 1:** Characteristics of participants.

Characteristics		Total (*n* = 6195)	
		*n* or Mean	% or SD
Maternal age (year)	Mean	31.4	4.8
	<25	460	7.4
	25–29	1721	27.8
	30–34	2325	37.5
	35–39	1405	22.7
	≥40	272	4.4
	Missing	12	0.2
Paternal age (year)	Mean	33.3	5.5
	<24	270	4.4
	25–29	1284	20.7
	30–34	2141	34.6
	35–39	1718	27.7
	≥40	763	12.3
	Missing	19	0.3
Age difference between partner and woman	<−5	118	1.9
	−5–−1	1224	19.8
	0–4	3609	58.3
	5–9	939	15.2
	10–14	218	3.5
	≥15	68	1.1
	Missing	19	0.3
Someone who supported during pregnancy	Yes	6042	97.5
	No	134	2.2
	Missing	19	0.3
Financial status	Stable	2784	44.9
	Able to manage	2502	40.4
	Difficult to manage or unstable	659	10.6
	Missing	250	4.0
Maternal employment status	Full-time	1027	16.6
	Part-time	295	4.8
	Not working	4794	77.4
	Missing	79	1.3
Paternal employment status	Full-time	6033	97.4
	Part-time	48	0.8
	Not working	50	0.8
	Missing	64	1.0
Living with grandfather	No	5673	91.6
	Yes	522	8.4
Living with grandmother	No	5513	89.0
	Yes	682	11.0
Infertility treatment	No	5399	87.2
	Yes	726	11.7
	Missing	70	1.1
Unintended pregnancy	No	5738	92.6
	Yes	443	7.2
	Missing	14	0.2
Sex of the child	Boy	3116	50.3
	Girl	3027	48.9
	Missing	52	0.8
Birth order	First child	3105	50.1
	Subsequent child	3075	49.6
	Missing	15	0.2
Unsatisfactory delivery	No	6038	97.5
	Yes	141	2.3
	Missing	16	0.3
Low birth weight	No	5655	91.3
	Yes	517	8.3
	Missing	23	0.4
Preterm	No	5725	92.4
	Yes	350	5.6
	Missing	120	1.9
Maternal smoking history	No	5570	89.9
	Yes	623	10.1
	Missing	2	0.0
Maternal drinking history	No	5948	96.0
	Yes	236	3.8
	Missing	11	0.2
History of miscarriage	No	5091	82.2
	Yes	1102	17.8
	Missing	2	0.0
Termination of pregnancy	No	5790	93.5
	Missing	403	6.5
	Yes	2	0.0
Verbal abuse from partner	None	5556	89.7
	A few times/sometimes/often	617	10.0
	Missing	22	0.4
Physical abuse from partner	None	6128	98.9
	A few times/sometimes/often	60	1.0
	Missing	7	0.1
Maternal depressive symptoms during one year prior to pregnancy	No	5317	85.8
	Yes	867	14.0
	Missing	11	0.2
Maternal history of mental disorders	No	6109	98.6
	Yes	86	1.4

**Table 2:** Social support and the Edinburgh Postnatal Depression Scale (EPDS) score.

Variables		Total (*n* = 6195)	
		*n*	%
Consult with partner	Yes	6016	97.1
	No	170	2.7
	Missing	9	0.1
Consult with others (at least one person out of parents, relatives, or friends close by for far)	Yes	6076	98.1
	No	119	1.9
Classification based on social support from partner or others	Having both partner’s and others’ social support	5908	95.4
	Having partner’s support, but not others’	108	1.7
	Having others’ support, but not partner’s	168	2.7
	Not having either partner’s or others’ support	11	0.2
EPDS score	≤8	5658	91.3
	≥9	536	8.7
	Missing	1	0.0

**Table 3:** Association between social support categories of mothers and PPD.

Variables		Prevalence of PPD (%)	Crude		Model 1		Model 2		Model 3		Model 4	
			OR	95% CI	OR	95% CI	OR	95% CI	OR	95% CI	OR	95% CI
Classification based on social support from partner or others	Having both partner’s and others’ social support	7.5	Ref		Ref		Ref		Ref		Ref	
	Having partner’s support, but not others’	21.3	**3.32**	**2.07–5.32**	**2.74**	**1.68–4.49**	**2.56**	**1.54–4.24**	**2.53**	**1.52–4.22**	**2.34**	**1.37–3.98**
	Having others’ support, but not partner’s	36.9	**7.18**	**5.17–9.97**	**4.75**	**3.35–6.73**	**4.23**	**2.94–6.08**	**3.13**	**2.15–4.56**	**3.13**	**2.11–4.63**
	Not having either partner’s or others’ support	63.6	**21.5**	**6.27–73.7**	**13.3**	**3.63–48.9**	**13.2**	**3.50–49.6**	**13.3**	**3.47–50.7**	**7.22**	**1.76–29.6**
Maternal age (year)	<25	16.1	**2.45**	**1.82–3.28**	**1.76**	**1.28–2.41**	**1.72**	**1.24–2.40**	**1.53**	**1.09–2.15**	**1.46**	**1.02–2.08**
	25–29	8.3	1.16	0.92–1.46	1.06	0.83–1.35	1.07	0.83–1.37	1.07	0.83–1.38	1.04	0.80–1.35
	30–34	7.2	Ref		Ref		Ref		Ref		Ref	
	35–39	7.9	1.09	0.85–1.40	1.11	0.85–1.43	1.05	0.80–1.37	1.07	0.81–1.40	1.10	0.83–1.45
	≥40	14.0	**2.07**	**1.42–3.02**	**1.99**	**1.33–2.97**	**1.67**	**1.1–2.53**	**1.77**	**1.16–2.69**	**1.78**	**1.15–2.76**
Age difference between partner and woman	<−5	11.0	1.44	0.78–2.65	1.23	0.64–2.33	1.28	0.67–2.45	1.22	0.63–2.35	1.06	0.53–2.11
	−5–0	7.9	Ref		Ref		Ref		Ref		Ref	
	0–5	8.4	1.06	0.84–1.35	1.07	0.83–1.37	1.07	0.83–1.38	1.08	0.84–1.40	1.08	0.83–1.41
	5–10	9.3	1.19	0.88–1.61	1.11	0.80–1.53	1.05	0.76–1.46	1.09	0.78–1.51	1.03	0.73–1.45
	10–15	11.5	1.50	0.94–2.40	1.35	0.82–2.22	1.24	0.74–2.06	1.29	0.77–2.17	1.24	0.72–2.12
	≥15	13.2	1.77	0.85–3.68	1.64	0.76–3.51	1.60	0.74–3.49	1.54	0.70–3.39	1.33	0.57–3.11
Someone who supported during pregnancy	Yes	8.4	Ref		Ref		Ref		Ref	0	Ref	
	No	18.7	**2.49**	**1.60–3.88**	1.52	0.92–2.52	1.39	0.83–2.32	1.35	0.81–2.26	1.14	0.67–1.94
Financial status	Stable	4.2	Ref		Ref		Ref		Ref		Ref	
	Able to manage	10.2	**2.58**	**2.06–3.23**	**2.35**	**1.87–2.95**	**2.20**	**1.74–2.78**	**2.16**	**1.71–2.73**	**2.12**	**1.67–2.70**
	Difficult to manage or unstable	21.1	**6.04**	**4.64–7.85**	**4.62**	**3.50–6.11**	**3.97**	**2.98–5.31**	**3.68**	**2.75–4.93**	**3.32**	**2.45–4.48**
Maternal employment status	Full-time	6.7	Ref		Ref		Ref		Ref		Ref	
	Part-time	11.9	**1.84**	**1.20–2.82**	1.24	0.79–1.95	1.15	0.72–1.84	1.14	0.71–1.83	1.05	0.64–1.72
	Not working	8.9	**1.34**	**1.03–1.74**	1.10	0.84–1.45	1.09	0.82–1.45	1.13	0.85–1.51	1.09	0.81–1.46
Paternal employment status	Full-time	8.5	Ref		Ref		Ref		Ref		Ref	
	Part-time	20.8	**2.83**	**1.40–5.70**	1.31	0.60–2.85	1.51	0.69–3.33	1.60	0.73–3.51	1.68	0.76–3.70
	Not working	20.0	**2.68**	**1.33–5.40**	1.77	0.84–3.74	1.70	0.77–3.72	1.64	0.73–3.67	1.82	0.78–4.25
Living with grandfather	No	8.5	Ref		Ref		Ref		Ref		Ref	
	Yes	9.8	1.16	0.85–1.57	0.83	0.51–1.36	0.89	0.54–1.47	0.93	0.56–1.55	1.04	0.62–1.75
Living with grandmother	No	8.3	Ref		Ref		Ref		Ref		Ref	
	Yes	11.1	**1.37**	**1.06–1.78**	1.42	0.94–2.16	1.34	0.87–2.05	1.25	0.81–1.93	1.18	0.75–1.86
Infertility treatment	No	8.7	Ref				Ref		Ref		Ref	
	Yes	7.7	0.87	0.65–1.16			1.12	0.81–1.54	1.13	0.82–1.55	1.15	0.82–1.59
Unintended pregnancy	No	7.5	Ref				Ref		Ref		Ref	
	Yes	23.5	**3.80**	**2.98–4.83**			**2.49**	**1.90–3.26**	**2.36**	**1.80–3.10**	**2.17**	**1.64–2.88**
Sex of the child	Boy	8.9	Ref				Ref		Ref		Ref	
	Girl	8.5	0.95	0.79–1.13			0.99	0.82–1.19	1.02	0.84–1.24	1.03	0.85–1.25
Birth order	First child	8.7	Ref				Ref		Ref		Ref	
	Subsequent child	8.6	0.99	0.83–1.18			0.96	0.78–1.17	0.92	0.75–1.12	1.01	0.82–1.24
Unsatisfactory delivery	No	8.1	Ref				Ref		Ref		Ref	
	Yes	33.3	**5.67**	**3.95–8.15**			**4.15**	**2.76–6.24**	**4.01**	**2.65–6.06**	**3.55**	**2.29–5.50**
Low birth weight	No	8.3	Ref				Ref		Ref		Ref	
	Yes	12.0	**1.50**	**1.13–1.98**			1.22	0.86–1.73	1.21	0.85–1.72	1.27	0.89–1.82
Preterm	No	8.3	Ref				Ref		Ref		Ref	
	Yes	12.6	**1.57**	**1.13–2.19**			1.09	0.72–1.66	1.09	0.71–1.65	1.09	0.71–1.68
Maternal smoking history	No	8.0	Ref				Ref		Ref		Ref	
	Yes	14.6	**1.97**	**1.54–2.50**			1.19	0.90–1.57	1.11	0.84–1.48	1.01	0.75–1.36
Maternal drinking history	No	8.4	Ref				Ref		Ref		Ref	
	Yes	13.1	**1.64**	**1.11–2.41**			1.37	0.90–2.10	1.33	0.87–2.04	1.23	0.79–1.93
History of miscarriage	No	8.6	Ref				Ref		Ref		Ref	
	Yes	9.0	1.05	0.83–1.32			1.10	0.86–1.41	1.09	0.85–1.40	1.13	0.87–1.46
Termination of pregnancy	No	8.4	Ref				Ref		Ref		Ref	
	Yes	12.9	**1.62**	**1.19–2.20**			1.13	0.81–1.59	1.06	0.75–1.50	0.98	0.68–1.41
Verbal abuse from partner	No	7.0	Ref						Ref		Ref	
	Yes	23.0	**3.95**	**3.19–4.89**					**2.33**	**1.81–2.99**	**1.89**	**1.45–2.46**
Physical abuse from partner	No	8.4	Ref						Ref		Ref	
	Yes	36.7	**6.34**	**3.72–10.8**					1.82	0.97–3.40	1.67	0.86–3.21
Maternal depressive symptoms during one year prior to pregnancy	No	5.7	Ref								Ref	
	Yes	26.2	**5.81**	**4.80–7.03**							**3.90**	**3.15–4.84**
Maternal history of mental disorders	No	8.2	Ref								Ref	
	Yes	41.9	**8.06**	**5.20–12.5**							**3.57**	**2.13–5.99**

Bold signifies *p* < 0.05.
